# Characterization of Mannoprotein Structural Diversity in Wine Yeast Species

**DOI:** 10.1021/acs.jafc.3c05742

**Published:** 2023-12-04

**Authors:** Carla Snyman, Julie Mekoue Nguela, Nathalie Sieczkowski, Benoit Divol, Matteo Marangon

**Affiliations:** † South African Grape and Wine Research Institute, Department of Viticulture and Oenology, Stellenbosch University, Private Bag X1, Matieland 7602, South Africa; ‡ Department of Agronomy, Food, Natural Resources, Animals and Environment (DAFNAE), University of Padova, Viale Dell’Università, 16, 35020 Legnaro, Padova, Italy; § Lallemand SAS, 19 rue des Briquetiers, BP 59, 31702 Blagnac, France; ∥ Interdepartmental Centre for Research in Viticulture and Enology (CIRVE), University of Padova, Via XXVIII Aprile 14, 31015 Conegliano, Italy

**Keywords:** mannoprotein, yeast, wine, protein identification, polysaccharide characterization

## Abstract

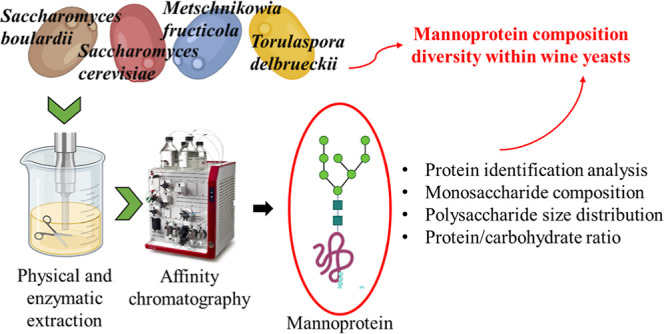

The structure of yeast cell wall (CW) mannoproteins (MPs) influences their impact on wine properties. Yeast species produce a diverse range of MPs, but the link between properties and specific structural features has been ill-characterized. This study compared the protein and polysaccharide moieties of MP-rich preparations from four strains of four different enologically relevant yeast species, named *Saccharomyces boulardii* (SB62), *Saccharomyces cerevisiae* (SC01), *Metschnikowia fructicola* (MF77), and *Torulaspora delbrueckii* (TD70), and a commercial MP preparation. Monosaccharide determination revealed that SB62 MPs contained the highest mannose/glucose ratio followed by SC01, while polysaccharide size distribution analyses showed maximum molecular weights ranging from 1349 kDa for MF77 to 483 kDa for TD70. Protein identification analysis led to the identification of unique CW proteins in SB62, SC01, and TD70, as well as some proteins shared between different strains. This study reveals MP composition diversity within wine yeasts and paves the way toward their industrial exploitation.

## Introduction

The diversity of fungal cell wall (CW) polysaccharide properties has led to their widespread application in numerous industries including cosmetics, medicine, agriculture, and food and beverage.^1^ For example, β-glucans and mannoproteins (MPs) extracted from the CW of the ascomycetous yeast *Saccharomyces cerevisiae* have been utilized or tested as nutraceuticals and additives in food products such as baked goods, confectionary, yogurt, mayonnaise, salad dressing, fruit juices, and wine due to their fiber-rich, prebiotic, rheological, emulsifying, and stabilizing properties, among others.^2^ This diversity in bioactive and techno-functional properties is largely due to differences in polysaccharide structural features and composition, which may be influenced by factors such as their method of preparation, fungal growth conditions, and species of origin.^3,4^

The structural diversity of MPs in particular has been shown to influence their impact as additives on food and beverage quality and especially on wine properties.^5,6^ This family of glycoproteins forms the second most abundant group of CW components in *S. cerevisiae*, alongside β-glucan, and is known to improve the aspects of wine quality such as protein and tartrate stability, astringency, color stability, and foaming properties.^7^ The glycan moiety is mainly composed of mannose, and the typical protein content ranges between 1 and 10%.^8^ While their molecular weight (MW) has been shown to vary between 5 and 800 kDa, the typical reported range is 50–500 kDa.^7^

Different MPs have also shown considerable structural variations in terms of their mannose/glucose ratios, presence of other monosaccharides such as galactose, carbohydrate content in proportion to protein and degree of glycan branching, and MW and charge distribution.^6,9−12^ Some of these variations have, furthermore, been shown to play a role in their impact on wine properties. For example, changes in the mannose/glucose ratio have been closely correlated with changes in their impact on wine properties such as protein haze formation and tannin aggregation.^13,14^ Furthermore, their binding ability toward wine phenolics, which has implications for wine astringency and color, has been shown to be dependent on MP MW, carbohydrate/protein ratio, and phosphorylation.^5,12,15^ However, the links between the structural features of MPs and their impacts on wine properties are still poorly understood.

The enological benefits of MPs are largely harnessed through their release from the yeast CW into wine during alcoholic fermentation and aging on the lees. Furthermore, their use as additives to wine has been allowed by the International Organization of Vine and Wine (OIV Resolution Oeno 26/2004) and the regulatory authorities of several countries, including the European Union [Regulation (EC) no. 2165/2005]. Indeed, in recent decades, a large variety of MP-rich products, such as yeast extracts obtained through various physical, chemical, and enzymatic extraction methods and purified to different degrees, have become commercially available to improve properties such as astringency, mouthfeel, color, and protein and tartrate stability. The extraction of MPs for exogenous application to wine provides a number of advantages over the reliance on their release from the yeast CW during wine production. Besides the microbiological and organoleptic risks involved, aging on the lees is a time- and resource-consuming practice.^16^ Extraction techniques furthermore provide the opportunity for the exploration and potential exploitation of MP diversity that exists between different yeast species that would normally have been outcompeted during the early stages of fermentation.

Indeed, MPs released from non-*Saccharomyces* yeasts have been shown to improve wine properties in different ways. For instance, whereas *Schizosaccharomyces pombe* and *Lachancea thermotolerans* MPs showed the ability to improve wine mouthfeel and aromatic characteristics, those from *Torulaspora delbrueckii* provided protein haze protection and color stabilization.^17,18^ Furthermore, some structural characteristics of MPs have been shown to vary between different yeast species and strains in terms of features such as their protein/carbohydrate ratio, monosaccharide composition, glucose/mannose ratio, and MW.^4,19,20^ Nevertheless, information regarding the structural diversity of MPs among yeast species remains limited.

This study sought to characterize and compare the structural features of MPs extracted and purified from strains from four different wine yeast species. Total sugar and protein yields were measured before analyzing the monosaccharide content and polysaccharide size distribution. A commercially available MP preparation (from *S. cerevisiae*) was included as a control. An evaluation of the proteins contained in the purified preparations was furthermore carried out through a protein identification (PID) analysis. The ultimate objective, of which this study forms the starting point, is understanding the influence of the species/strains of origin on MP structure and composition and, in turn, on their impact on wine properties, thus contributing to a clearer picture of the structure–function relationship of MPs and those factors involved in their diversity.

## Materials and Methods

### Extraction and Isolation of MPs

#### Physical and Enzymatic Extraction

MPs were extracted from liquid cultures of the following strains: *Saccharomyces boulardii* (SB62), *Saccharomyces cerevisiae* (SC01), *Metschnikowia fructicola* (MF77), and *T. delbrueckii* (TD70) obtained from the yeast culture collection of Lallemand Inc. (Montreal, QC, Canada). Yeast strains were cultivated in an enrichment medium [yeast extract (10 g/L), peptone (20 g/L), and glucose (20 g/L)] prepared in 0.1 M McIlvaine’s buffer adjusted to pH 5 and cultured at 30 °C with shaking at 120 rpm, as described by Snyman et al.^21^ (2021), and cells were collected through centrifugation after 48 h of incubation. Cells were subsequently resuspended in 0.1 M phosphate buffer, pH 6.5, to a volume of 200 mL at a concentration of 2.5 × 10^8^ cells/mL. The beaker containing the suspension was placed on ice for the duration of ultrasound treatment using a horn-type sonicator (Sonopuls GM 200 apparatus, Bandelin, Germany) equipped with a 6 mm probe. The suspension was sonicated with a 50% duty cycle (the percentage total treatment time in which sonication was occurring) using 30 s pulses, 50% amplitude (the percentage of the maximum amplitude that can be delivered by the sonicator), and for a total sonication duration of 4 min.

After the ultrasound treatment, samples were centrifuged at 4500*g* for 10 min, and the supernatant was discarded. The pellet was resuspended in phosphate buffer to a volume of 50 mL in a 100 mL Erlenmeyer flask. To this suspension was added lyticase from *Arthrobacter luteus* (β-1,3-glucanase, Sigma-Aldrich, St Louis, MO) at a concentration of 1000 units of enzyme per gram of dry weight of cells. The enzymatic treatment was carried out at 37 °C for 20 h with shaking at 60 rpm and thereafter inactivated at 60 °C for 10 min. After centrifugation at 2000*g* for 10 min, the supernatant was collected and filtered through a 0.45 μM syringe filter.

#### MP Purification

MPs extracted after ultrasound and enzymatic treatments were purified through fast protein liquid chromatography (FPLC) using an ÄKTA purifier 10 FPLC apparatus (GE Healthcare, Milan, Italy). Filtered supernatants were loaded at a flow rate of 0.5 mL/min onto a XK 26/40 column (GE Healthcare) containing 100 mL of concanavalin A (ConA) Sepharose 4B (Cytiva Europe, Milan, Italy) that had previously been equilibrated with binding buffer (20 mM Tris-HCl, pH 7.4, containing 0.5 M NaCl, 1 mM CaCl_2_, and 1 mM MnCl_2_). The unbound material was eluted as flow-through fractions with the binding buffer (approximately 4 column volumes) at a flow rate of 2 mL/min. The fraction retained by the ConA column was eluted with elution buffer [20 mM Tris-HCl, pH 7.4, containing 0.5 M NaCl and 0.2 M methyl-α-d-mannopyranoside (Merck, Milan, Italy) at a flow rate of 2 mL/min (eluted fraction)]. Eluted MPs were detected by monitoring the absorbance at 280 nm and collected for dialysis with a Spectra/Por 3.5 kDa cutoff membrane (Spectrum Laboratories Inc., Eindhoven, The Netherlands) against distilled water for 24 h at 4 °C. Dialyzed fractions were freeze-dried and weighed before downstream analyses were performed.

### Structural Characterization of MPs

#### Total Sugar Quantification

Total sugar content in freeze-dried MP preparations was determined using the phenol sulfuric acid test, estimated from a standard curve constructed from mannose as described previously.^21^ Freeze-dried MPs and a commercially available MP, hereinafter referred to as LMP, were resuspended in deionized water produced by a Milli-Q system (Merck Millipore, Darmstadt, Germany) at a concentration of 2 mg/mL. In a 96-well microplate, 150 μL of sulfuric acid was added to a 50 μL sample or mannose to which 30 μL of phenol (5% w/v) was added, and the plate was incubated at 30 °C for 20 min. Colorimetric detection of sugars was performed by measuring the absorbance at 490 nm using a Thermo Scientific Multiskan GO Microplate spectrophotometer with SkanIt software.

#### Protein Quantification

Freeze-dried MPs, as well as the commercially available MP LMP, were resuspended in Milli-Q water at a concentration of 5 mg/mL before the determination of total protein content using the Pierce BCA protein assay kit (Thermo Scientific, Waltham, MA, USA) according to the manufacturer’s instructions. Colorimetric detection of proteins was performed by measuring the absorbance at 562 nm.

#### Carbohydrate and Protein Visualization

Sodium dodecyl sulfate polyacrylamide gel electrophoresis (SDS–PAGE) and native PAGE were used to visualize carbohydrates and proteins in this study. Freeze-dried MP samples were prepared by resuspending in Milli-Q water to a concentration of 1 mg/mL. MP samples that had been deglycosylated (described below) were also visualized.

SDS–PAGE was performed as previously described.^22^ Gels containing 15% bis-acrylamide were loaded with the samples as described above, which had been diluted with a loading buffer to reach final concentrations of 17.5 mM Tris-HCl (pH 6.8), 0.8% SDS (w/v), 9% glycerol (v/v), 2.5% β-mercaptoethanol (w/v), and 0.002% bromophenol blue (w/v). Electrode chambers were filled with running buffer [50 mM Tris, 200 mM glycine, and 0.2% SDS (w/v)]. Native PAGE was performed with gels cast without the addition of SDS [resolving gel: 375 mM Tris-HCl pH 8.8, 15% bis-acrylamide (w/v), 0.05% ammonium persulfate (APS) (w/v), and 0.05% *N*,*N*,*N*′,*N*′-tetramethylethylenediamine (TEMED) (v/v); stacking gel: 125 mM Tris-HCl pH 6.8, 4% bis-acrylamide (w/v), 0.05% APS (w/v), and 0.4% TEMED (v/v)] and with loading buffer and running buffer prepared as described above but without the addition of SDS. Gels were electrophoresed on a Bio-Rad Mini-Protean Tetra cell system (Bio-Rad Laboratories, Hercules, CA, USA).

For carbohydrate visualization, gels were stained using the periodic acid-Schiff (PAS) procedure described previously.^23^ Coomassie staining was performed for the visualization of proteins, in which gels were stained overnight in staining solution [1 g Coomassie blue R250 (Merck, Darmstadt, Germany) in 50% (v/v) ethanol and 10% (v/v) acetic acid] and destained with 12.5% isopropanol and 10% (v/v) acetic acid. Images of the gels were captured using a Molecular Imager Gel Doc system (Bio-Rad Laboratories) with Image Lab software v6.0 (Bio-Rad Laboratories).

#### Monosaccharide Determination

The mannose and glucose composition of the freeze-dried MPs and the commercial LMP was determined using a gas chromatography–flame ionization detection (GC–FID) method.^24^ MPs were suspended in 2 M trifluoroacetic acid (TFA) (Sigma-Aldrich) to a concentration of 2 mg/mL and allowed to incubate at 110 °C for 2 h. The reaction was centrifuged at 15,000*g* for 5 min, and the excess reagent was removed from the supernatant under a stream of nitrogen gas at 60 °C. Acidic methanolysis was performed by adding 500 μL of methanol/3 M HCl: dry methanol [1:2 (v/v)] to the desiccated sample and incubating for 16 h at 80 °C. The reaction was then dried under nitrogen gas at 40 °C, and another 250 μL of dry methanol was added. This step was repeated before a final desiccation under nitrogen gas at 40 °C. The obtained methyl glycosides were converted to their trimethylsilyl (TMS) derivatives following the addition of 150 μL of a mix of hexamethyldisilane: chlorotrimethylsilane: pyridine [2:1:10 (v/v)] (silylating mixture I according to Sweeley, Sigma-Aldrich) and a 20 min incubation at 80 °C. The reagent was removed under nitrogen gas at 80 °C, and 1 mL of cyclohexane was added before analysis with GC–FID. Myo-inositol (Sigma-Aldrich) was used as an internal standard, and standards of mannose and glucose were similarly derivatized and analyzed to obtain patterns for identification and for the construction of standard calibration curves. All reactions were carried out in triplicate.

Separation of the monosaccharides was performed on a gas chromatograph (Trace 1200, Thermo Scientific) with a nonpolar ZB-5MS (30 m, 0.25 mm ID, 0.25 μm film thickness) capillary column (Phenomenex, Torrance, CA, USA). Hydrogen was used as the carrier gas at a flow rate of 1 mL/min. The injector temperature was maintained at 250 °C, and 1 μL of sample was injected in splitless mode. The oven temperature was programmed as follows: 80 °C for 1 min, ramped up to 300 °C at a rate of 7 °C/min, and held for 2 min.

#### Polysaccharide Size Distribution Analysis

The concentration and MW distribution of polysaccharides in the freeze-dried MP samples and the commercial LMP were determined using a high-resolution size-exclusion chromatography (HRSEC) method.^25,49^ Samples were resuspended in running buffer (50 mM ammonium formate) to reach a concentration of 1 mg/mL. After centrifugation at 14,000*g* for 2 min, the supernatant was transferred to HPLC vials. Analyses were performed using an Agilent 1260 series II quaternary pump LC (Agilent Technologies, Milan, Italy) equipped with an RI (refractive index) detector. Before injection of 100 μL into the system, samples were held at 8 °C in a temperature-controlled autosampler. Separation was performed on a gel permeation HPLC column (PL-Aquagel-OH 50, Agilent) at room temperature. The mobile phase was applied at a constant flow rate of 0.6 mL/min for 35 min, and the temperature of the RID cell was kept at 35 °C. A qualitative calibration curve made with 10 pullulan standards (Merk, Darmstadt, Germany) of MW ranging between 342 and 805,000 Da was used for the determination of MP MW distribution. Polysaccharide quantification was performed by using a calibration curve constructed with pectin and dextran in the range between 0 and 2 g/L.

#### PID Analysis

Prior to PID analysis of the protein moiety of freeze-dried MPs, samples were subjected to a deglycosylation reaction using PNGase F (Peptide: N-glycosidase F, New England BioLabs, Ipswich, MA, USA) according to the manufacturer’s instructions. Deglycosylated MPs were visualized using SDS–PAGE and Coomassie staining as described above. Selected protein bands were excised from the gels and sequenced by LC–MS/MS after trypsin in-gel digestion at the Centre for Proteomic and Genomic Research (CPGR, Cape Town, South Africa). Proteins were identified through peptide spectrum matches (PSMs) after database interrogation performed with Byonic software v3.8.13 (Protein Metrics, CA, USA) using the proteomes of *S. cerevisiae* ATCC 204508/S288c (UP000002311) for MF77 and TD70, *S. cerevisiae* Lalvin EC1118 (UP000000286) for SC01, and *S. boulardii* (UP000037662) for SB62, sourced from UniProt. Proteins identified within each MW range for the different MPs were ranked according to |Log Prob| [log base 10 of the protein *p*-value, which is the likelihood of the PSMs to this protein (or protein group) arising by random chance] to which a cutoff value of 4 was applied.

## Results

### MP Purification

**Figure 1:**
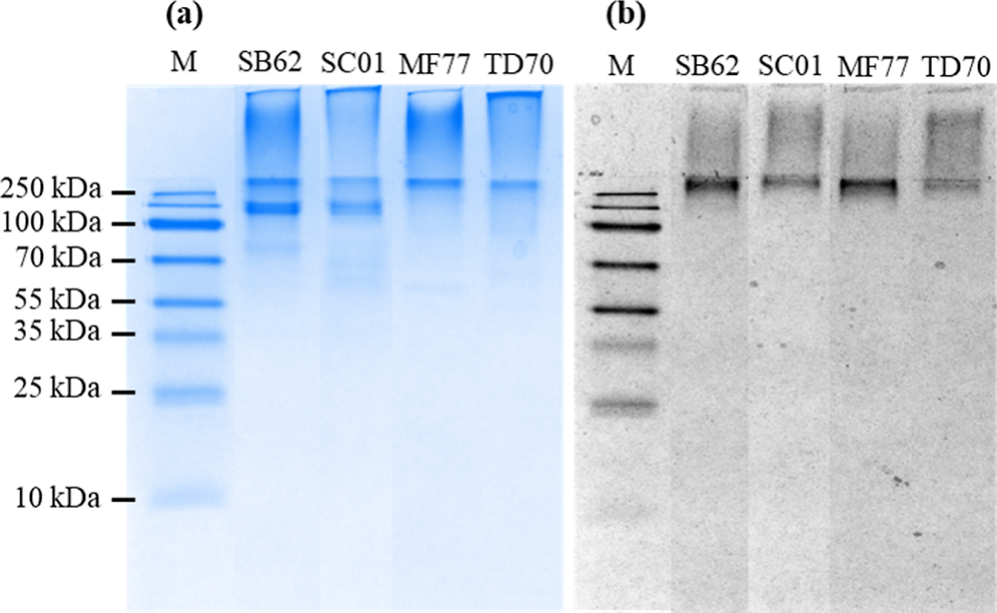
Native PAGE visualization of freeze-dried purified MPs from *S. boulardii* (SB62), *S. cerevisiae* (SC01), *M. fructicola* (MF77), and *T. delbrueckii* (TD70). Gels were stained with (a) Coomassie for the visualization of proteins or (b) Schiff’s reagent for the visualization of carbohydrates. M: molecular weight marker (PageRuler Plus Prestained Protein Ladder, Thermo Scientific).

Eluted fractions containing purified MPs were collected and pooled together before dialysis (3.5 kDa of MWCO) and freeze-drying. Freeze-dried MPs were subsequently visualized after native PAGE and staining with Coomassie blue or Schiff’s reagent (Figure 1a,b, respectively). Low-mobility protein bands (MW > 250 kDa) were visible for all MP samples after Coomassie staining, as well as a smear in the stacking gel. Additionally, two protein bands of similar mobility were evident at ∼125 kDa for SB62 and SC01 (lanes 1 and 2, respectively). Faint protein bands were also detected at ∼68 and ∼63 kDa for SC01, at ∼79 kDa for SB62, and at ∼60 kDa for MF77 (lane 3). Schiff-stained smears in the stacking gel and a low-mobility (MW > 250 kDa) carbohydrate band were visible for all MPs, as shown in Figure 1b.

**Figure 2:**
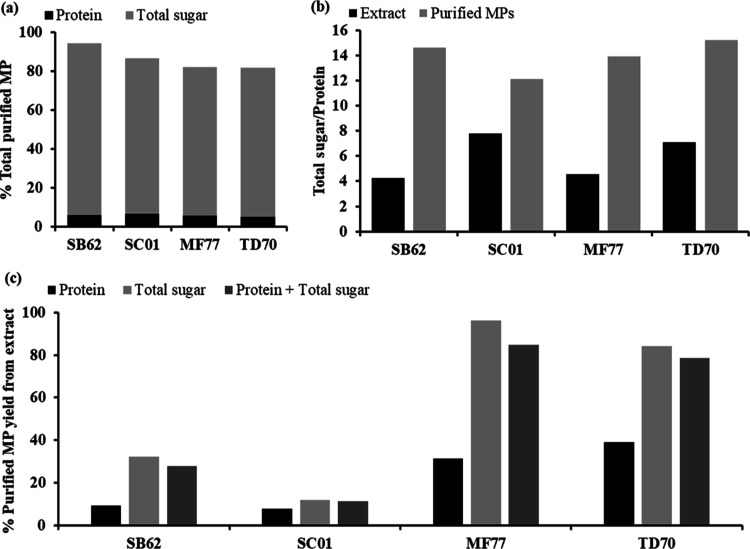
Protein and sugar yields obtained in crude extracts and purified MPs derived from cultures of *S. boulardii* (SB62), *S. cerevisiae* (SC01), *M. fructicola* (MF77), and *T. delbrueckii* (TD70). (a) Protein and sugar content as a percentage of the dry weight of purified MPs. (b) Sugar-to-protein ratios obtained in purified MPs and in crude extracts. (c) Percentage yield of protein, sugar, and combined protein + sugar in purified MPs from crude extracts.

After quantification, the protein and total sugar yields in crude MP extracts and purified MPs after freeze-drying were compared for SB62, SC01, MF77, and TD70 (Figure 2). The purified protein yield from the extract for MF77 and TD70 was 22.1–31.4% higher than that for SB62 and SC01 (Figure 2c). Total sugar yields were similarly higher in MF77 and TD70, with a 51.8–84.4% increase compared to SB62 and SC01 (Figure 2c). Furthermore, purified sugar yields from the crude extract were higher for all MPs compared to purified protein yields, from 4.2% higher for SC01 to 64.8% for MF77 (Figure 2c). Thus, the combined protein + sugar purified yields normalized by extract yields were lower than sugar only but higher than protein. It follows that the sugar/protein ratio increased for all MPs after purification and showed 12.1–15.2-fold higher levels of sugars than proteins (Figure 2b). Whereas SC01 showed the smallest fold increase from the crude extract to purified MP at 4.3, the ratio of sugar/protein increased 10.4-fold in purified SB62. Combined protein and total sugar yield from crude extracts followed a similar trend for all MPs as when protein and sugar were taken alone and were recorded as 27.99, 11.45, 84.66, and 78.57% for SB62, SC01, MF77, and TD70, respectively (Figure 2c).

The percentage composition of sugar and protein contained in freeze-dried MPs was further calculated (Figure 2a). Protein varied slightly for MF77 and TD70 at 5.5 and 5.0%, respectively, and SB62 and SC01 at 6.0 and 6.6%, respectively. However, sugar composition in purified SB62 at 88.2% was 8.4–11.7% higher than all other MPs.

### Monosaccharide Composition

**Figure 3:**
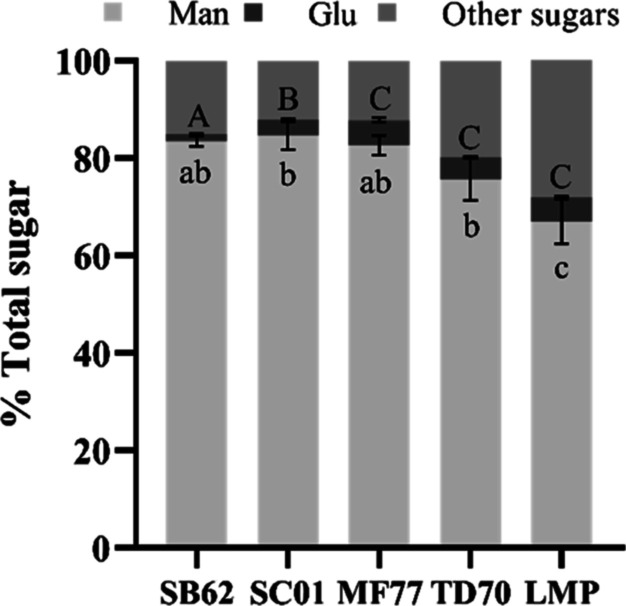
Mannose (Man) and glucose (Glu) compositions as a percentage of the total sugar in purified MPs from *S. boulardii* (SB62), *S. cerevisiae* (SC01), *M. fructicola* (MF77), and *T. delbrueckii* (TD70) as well as in a commercial MP product (LMP). “Other sugars” indicates the proportion of monosaccharides not accounted for by mannose and glucose. The data points shown are the means for three independently derivatized samples, and the error bars indicate the standard deviation between triplicates. Different upper and lower case letters indicate significant differences in glucose and mannose composition, respectively, between samples (*p* < 0.05) as analyzed independently by one-way ANOVA and the Fisher’s LSD test.

The monosaccharide components of all MPs were analyzed through GC–FID, particularly glucose and mannose. Figure 3 presents the percentage composition of monosaccharides obtained through normalization of monosaccharide concentrations with the total sugar measured through the phenol sulfuric acid assay. Percentage mannose ranged from 67% for LMP and 76% for TD70 to 83–85% for SB62, SC01, and MF77, whereas glucose comprised 1.6, 3.4, 5.2, 4.5, and 5.0% of the total sugars in SB62, SC01, MF77, TD70, and LMP, respectively. Thus, the ratio of mannose/glucose was highest for SB62 at 52.1 followed by SC01 at 24.9, whereas MF77, TD70, and LMP ranged from 13.4 to 16.8.

### Polysaccharide Size Distribution

**Figure 4:**
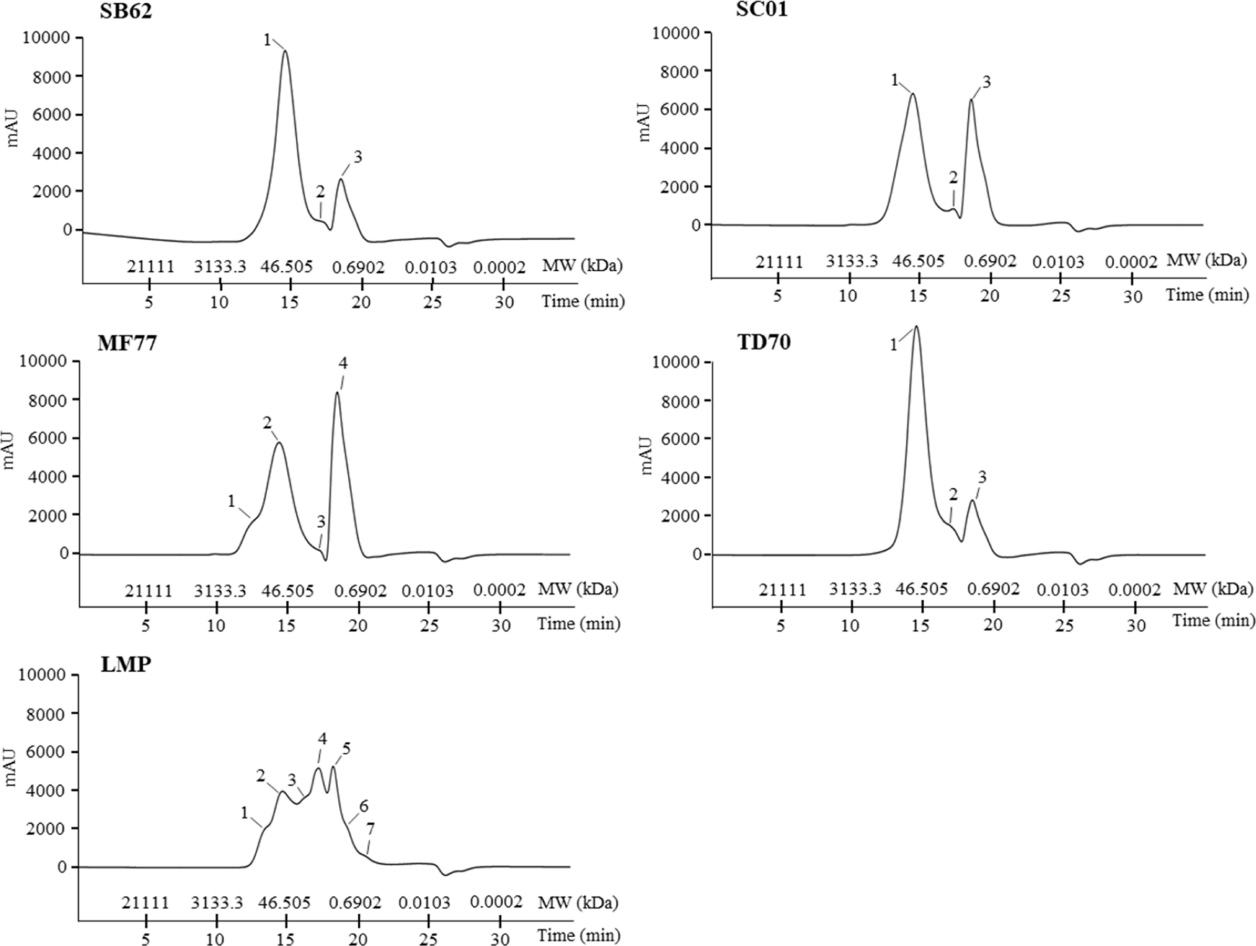
HRSEC profiles of polysaccharides in MPs purified from *S. boulardii* (SB62), *S. cerevisiae* (SC01), *M. fructicola* (MF77), and *T. delbrueckii* (TD70) as well as in a commercial MP product (LMP). The numbering of peaks for each MP is also shown and elaborated upon in Table 1.

Purified MPs and the commercial LMP formulation were subjected to HRSEC analysis for the characterization of their polysaccharide MW distribution. The polysaccharide profiles obtained are depicted by the chromatographs presented in Figure 4. Three peaks each were identified for SB62, SC01, and TD70, whereas a fourth peak was observed in the profile of MF77 and seven in that of LMP. The specific MW distribution characteristics of these peaks are described in Table 1. Peaks designated 2 and 3 for SB62, SC01, and TD70; 3 and 4 for MF77; and 4, 5, 6, and 7 for LMP likely indicate the presence of oligosaccharides in the MW range of 0.22–12.7 kDa. The profiles of SB62, SC01, MF77, and TD70 show similarity in terms of peak characteristics with the greatest differences being the MW range covered by >8 kDa polysaccharides, the relative concentration of polysaccharides compared to oligosaccharides, and the additional peak assigned to the profile of MF77. Indeed, whereas SB62 and SC01 showed similar maximum MWs of 1014 and 1022 kDa, respectively, MF77 reached up to 1349 kDa while TD70 polysaccharides were not larger than 483 kDa. Furthermore, >8 kDa polysaccharides formed 79.1 and 80.1% of the total poly- and oligosaccharides detected in SB62 and TD70, respectively, whereas this proportion decreased to 62.5% in SC01 and 57.1% in MF77. The additional high MW peak of 265 kDa detected in MF77 exceeded the highest MW peaks of SB62, SC01, and TD70 at 69.2, 77.7, and 67.7 kDa, respectively. Nevertheless, the weighted average MW of poly- and oligosaccharides for these four MPs was similar, falling in the range of 49.5–60.0 kDa. On the other hand, the weighted average MW for LMP was lower, at 32.5 kDa, and the size distribution profile for this MP was distinct from the other four profiles. The maximum MW of >12.7 kDa polysaccharides was 587 kDa, and the relative concentration of polysaccharides was 48.7% of the total poly- and oligosaccharides detected. However, the >12.7 kDa peak of the highest relative concentration showed a similar MW to that of the other MPs at 65.2 kDa.

**Table 1:** MW Distribution Characteristics of Polysaccharides in MPs Purified from *S. boulardii* (SB62), *S. cerevisiae* (SC01), *M. fructicola* (MF77), and *T. delbrueckii* (TD70) as Well as in a Commercial MP Product (LMP) Obtained by HRSEC

sample	peak ID^a^	MW range^b^ (kDa)	pMW^c^ (kDa)	relative concentration^d^ (%)	mMW^e^ (kDa)
LMP	1	139–587	139 ± 0.38	7.5 ± 0.08	32.5 ± 0.11
	2	28.3–139	65.2 ± 0.29	27.1 ± 0.08	
	3	12.7–28.3	12.8 ± 0.04	14.1 ± 0.08	
	4	4.6–12.7	7.6 ± 0.00	23.4 ± 0.06	
	5	1.8–4.59	3.2 ± 0.01	19.4 ± 0.14	
	6	0.7–1.77	1.8 ± 0.00	6.9 ± 0.04	
	7	0.2–0.68	0.7 ± 0.00	1.6 ± 0.12	
SB62	1	10.2–1014	69.2 ± 0.03	79.1 ± 0.17	55.5 ± 0.03
	2	4.7–10.2	10.2 ± 0.06	3.6 ± 0.05	
	3	0.5–4.71	2.5 ± 0.01	17.3 ± 0.15	
SC01	1	10.1–1022	77.7 ± 0.11	62.5 ± 0.05	49.5 ± 0.07
	2	4.6–10.1	7.0 ± 0.03	2.8 ± 0.03	
	3	0.3–4.6	2.4 ± 0.00	34.7 ± 0.07	
MF77	1	264–1349	265 ± 0.78	7.6 ± 0.11	60.0 ± 0.08
	2	8.1–264	78.4 ± 0.14	49.5 ± 0.08	
	3	4.9–8.14	8.1 ± 0.04	0.8 ± 0.04	
	4	0.6–4.95	2.5 ± 0.00	42.1 ± 0.03	
TD70	1	11.5–483	67.7 ± 0.09	80.1 ± 0.29	55.2 ± 0.07
	2	4.7–11.5	11.5 ± 0.01	5.1 ± 0.03	
	3	0.5–4.67	2.4 ± 0.01	14.7 ± 0.28	

a Numbering of peaks as shown in Figure 4.

b The upper and lower limits of MW for each peak.

c Peak MW. Mean ± SD (*n* = 3).

d Calculated on the basis of total polysaccharides. Mean ± SD (*n* = 3).

e Weighted average MW. Mean ± SD (*n* = 3).

### PID Analysis

**Figure 5:**
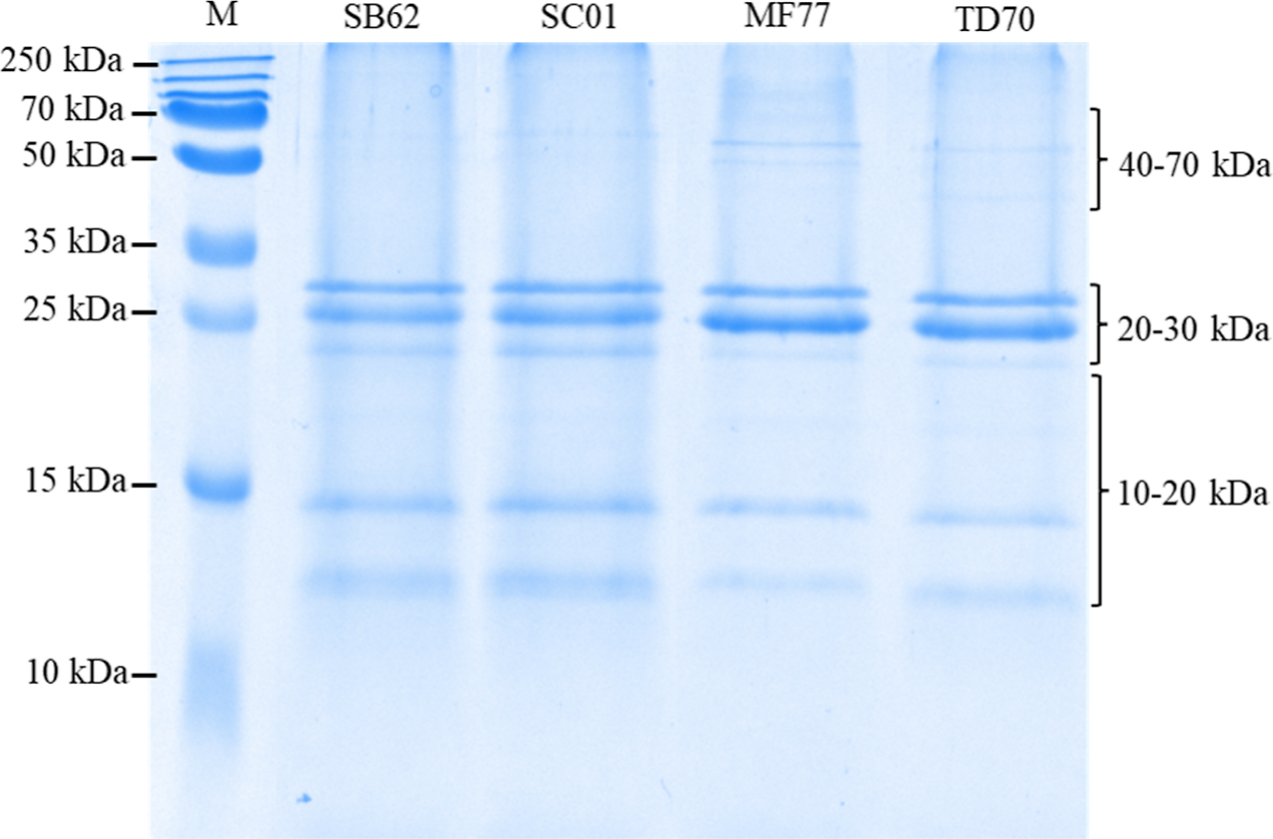
SDS–PAGE visualization of deglycosylated proteins from MPs purified from *S. boulardii* (SB62), *S. cerevisiae* (SC01), *M. fucticola* (MF77), and *T. delbrueckii* (TD70) after Coomassie blue staining. The MW ranges on the right of the gel indicate protein bands which were excised together for PID analysis. M: molecular weight marker (PageRuler Plus Prestained Protein Ladder, Thermo Scientific).

**Figure 6:**
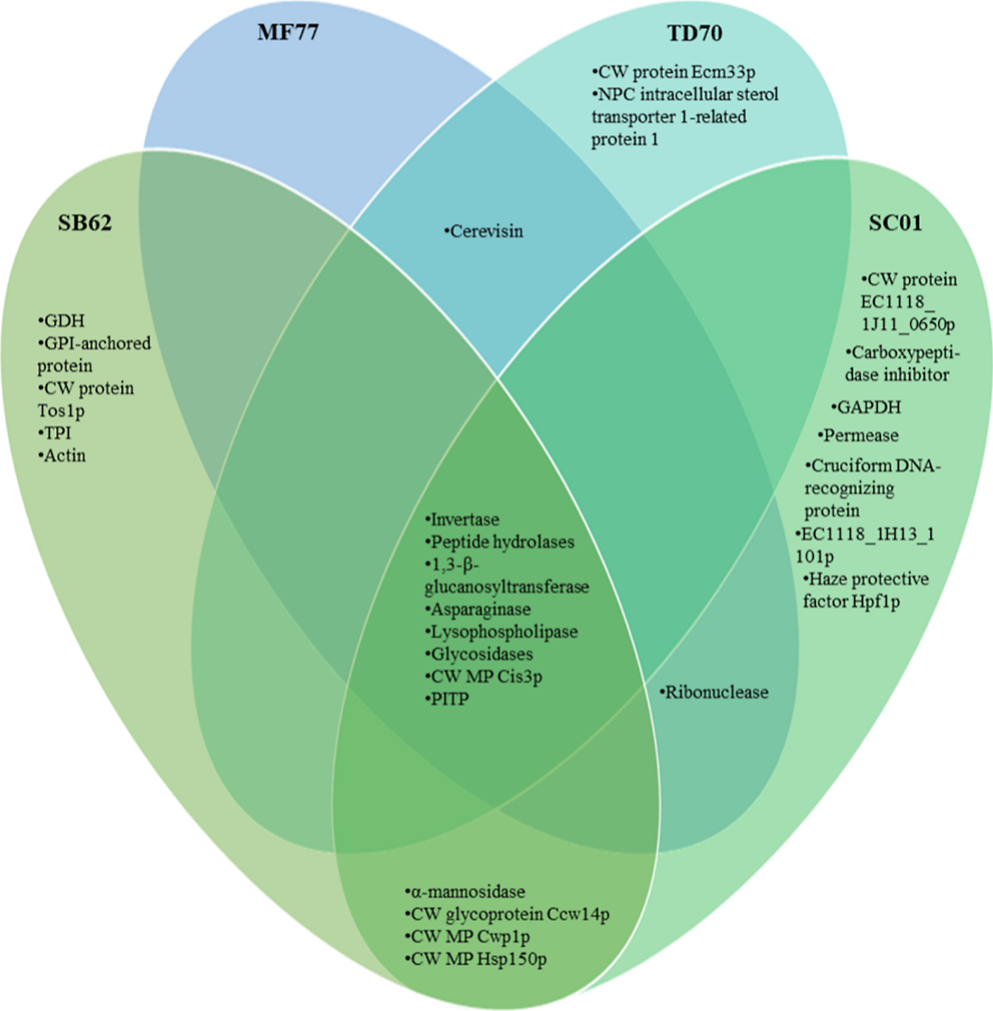
Similarities and differences between proteins identified at all MW ranges, excluding duplicates, through tandem mass spectrometry and Byonic software (listed in Table 2) in MPs from *S. boulardii* (SB62), *S. cerevisiae* (SC01), *M. fucticola* (MF77), and *T. delbrueckii* (TD70), represented in a Venn diagram. Peptide hydrolases include the identified exo- and endopeptidases. Glycosidases include glucan 1,3-β-glucosidase. Invertase also refers to saccharase. GDH: glutamate dehydrogenase. CW: cell wall. MP: mannoprotein. TPI: triosephosphate isomerase. GAPDH: glyceraldehyde-3-phosphate dehydrogenase. PITP: phosphatidylglycerol/phosphatidylinositol transfer protein.

PID analysis through LC–MS/MS sequencing and proteome database interrogation was carried out on excised protein bands stained by Coomassie blue after SDS–PAGE of deglycosylated MPs from SB62, SC01, MF77, and TD70. Protein bands included in three different MW ranges for each MP were analyzed independently, namely, 10–20, 20–30, and 40–70 kDa (Figure 5). Due to the low annotation scores of the reference proteome available for *T. delbrueckii* and the lack of a proteome database for *M. fructicola*, the reference proteome database of *S. cerevisiae* ATCC 204508/S288c was interrogated for the identification of proteins from MF77 and TD70. The proteins identified within each MW range for the different MPs with a |Log Prob| value above 4 are listed in Table 2, along with the best Byonic score of a PSM for the given protein. Additional information, including the UniProt entry names, |Log Prob| values, number of unique peptides, and percentage coverage, can be found in the Supporting Information (Table S1). Figure 6 displays a Venn diagram summarizing the global similarities and differences between MPs over all MW ranges.

**Table 2:** Proteins Identified through PSMs Using Tandem Mass Spectrometry and Byonic Software in MPs Purified from *S. boulardii* (SB62), *S. cerevisiae* (SC01), *M. fucticola* (MF77), and *T. delbrueckii* (TD70)^a^

	MW range^b^					
	40–70 kDa		20–30 kDa		10–20 kDa	
sample	protein name^c^	best score^d^	protein name^c^	best score^d^	protein name^c^	best score^d^
SB62	peptide hydrolase	783.7	*Bgl2pendobeta-1,3-glucanase*	946.5	Bgl2p *endobeta-1,3-glucanase*	547.9
	*saccharase*	649	peptide hydrolase	792.6	*saccharase*	375.7
	*1,3-beta-glucanosyltransfer-ase*	751.5	*saccharase*	563.7	*Cis3pmannose-containingglycoprotein constituent of the cell wall*	465.9
	*1,3-beta-glucanosyltransfer-ase*	531.6	*glycosidase*	684.4	*lysophospholipase*	304.4
	*glycosidase*	785.2	*Ygp1p cellwall-relatedsecretory glycoprotein*	553.4	peptide hydrolase	485.1
	APE1p aminopeptidase	853.7	*lysophospholipase*	537.5	*Hsp150pO-mannosylatedheat shock protein*	545
	*lysophospholipase*	562.2	*Cwp1p cell wall mannoprotein that localizes to the birth scars of daughter cells*	512.2	phosphatidylglycerol/phosphatidylinositol transfer protein	282
	Bgl2p *endobeta-1,3-glucanase*	610.4	Prb1p vacuolar proteinase B (YscB) with H3 N-terminal endopeptidase activity	501.7	*1,3-beta-glucanosyltransfer-ase*	420.7
	alpha-mannosidase	429.1	*Cis3pmannose-containingglycoprotein constituent of the cell wall*	633.1	actin	280.5
	*Ccw14p covalently linked cell wall glycoprotein*	644.1	*Tos1p covalently bound cell wall protein*	626.9	*glycosidase*	265.7
	*Ygp1p cellwall-relatedsecretory glycoprotein*	460.4	*1,3-beta-glucanosyltransfer-ase*	383		
	glutamate dehydrogenase (GDH)	390.8	*1,3-beta-glucanosyltransfer-ase*	515.2		
	*Cis3pmannose-containingglycoprotein constituent of the cell wall*	545	triosephosphate isomerase (TPI)	301.5		
	*GPI-anchoredprotein*	588.2	*1,3-beta-glucanosyltransfer-ase*	351.3		
	*1,3-beta-glucanosyltransfer-ase*	438.9	Ape4p cytoplasmic aspartyl aminopeptidase with possible vacuole function	315.8		
			phosphatidylglycerol/phosphatidylinositol transfer protein	229.1		
SC01	*saccharase*	598.9	Pep4p (proteinase)	774.8	Pep4p (proteinase)	852.9
	peptide hydrolase	674	*Bgl2p (glucan1,3-beta-glucosidase)*	1033.8	*Bgl2p (glucan1,3-beta-glucosidase)*	899.7
	Pep4p (proteinase)	891.2	*Cwp1p (cell wall protein)*	830.7	*Cwp1p (cell wall protein)*	810.6
	*1,3-beta-glucanosyltransfer-ase*	496.2	peptide hydrolase	771.5	*saccharase*	596.1
	*Cwp1p (cell wall protein)*	734.5	*saccharase*	632.3	peptide hydrolase	644.6
	*Bgl2p (glucan1,3-beta-glucosidase)*	710.1	*1,3-beta-glucanosyltransfer-ase*	664.1	*1,3-beta-glucanosyltransfer-ase*	731.9
	*Ygp1p (asparaginase)*	557.9	*Ygp1p (asparaginase)*	698.1	*Ygp1p (asparaginase)*	686.2
	*1,3-beta-glucanosyltransfer-ase*	678.3	Prb1p (proteinase)	817.3	*1,3-beta-glucanosyltransfer-ase*	561.8
	carboxypeptidase	599	*Exg1p (glucan1,3-beta-glucosidase)*	579.4	*lysophospholipase*	671.9
	*lysophospholipase*	540.9	*1,3-beta-glucanosyltransfer-ase*	539.8	Prb1p (proteinase)	694.5
	*EC1118_1J11_0650p (cell wall protein)*	455.3	*lysophospholipase*	641.5	*Exg1p (glucan1,3-beta-glucosidase)*	609.9
	Prb1p (proteinase)	532.6	*Cis3p (cell wall mannoprotein)*	728.7	cruciform DNA-recognizing protein 1	586.3
	*glycosidase*	472.3	*1,3-beta-glucanosyltransfer-ase*	624.3	*glycosidase*	483
	*Exg1p (glucan1,3-beta-glucosidase)*	456.6	*glycosidase*	554.2	*Cis3p (cell wall mannoprotein)*	841.9
	*1,3-beta-glucanosyltransfer-ase*	509.4	Tfs1p (carboxypeptidase inhibitor)	416.9	EC1118_1H13_1101p (uncharacterized protein)	523.9
	*Ccw14p (covalently linked cell wall protein)*	670.7	glyceraldehyde-3-phosphate dehydrogenase	639	*1,3-beta-glucanosyltransfer-ase*	580.3
	*Cis3p (cell wall mannoprotein)*	553.3	carboxypeptidase	471	phosphatidylglycerol/phosphatidylinositol transfer protein	494.1
	Lap4p (aminopeptidase)	319	*Adp1p (permease)*	344.2	*Hsp150p (cell wall mannoprotein)*	460
	*Ecm14p (peptidase)*	295.7	ribonuclease T(2)	482	carboxypeptidase	454.6
	alpha-mannosidase	296.9	phosphatidylglycerol/phosphatidylinositol transfer protein	512.5	*Hpf1p (haze protective factor)*	319.3
MF77	*invertase 2*	491	*glucan 1,3-beta-glucosidase*	700.5	*glucan1,3-beta-glucosidase*	461.2
	*1,3-beta-glucanosyltransfer-aseGAS1*	481.9	*invertase 2*	466	*invertase 2*	386.9
	*probable1,3-beta-glucanosyltransfer-aseGas3p*	529.1	cerevisin	615.3	aminopeptidase Y	536
	*probable glycosidase Crh1p*	414.7	*probable glycosidase CRH1*	395	*lysophospholipase 1*	688.8
	*glucan1,3-beta-glucosidase*	486.2	*cell wall mannoprotein Cis3p*	445.7	probable *1,3-beta-glucanosyltransfer-aseGas3p*	470.9
	*lysophospholipase 1*	428.1	*protein Ygp1p (asparaginase)*	402.3	phosphatidylglycerol/phosphatidylinositol transfer protein	403.7
	aminopeptidase Y	441	aminopeptidase Y	368.7	*1,3-beta-glucanosyltransfer-aseGas1p*	364.1
	*cell wall mannoprotein Cis3p*	423.9	*lysophospholipase 1*	395.8	*cell wall mannoprotein Cis3p*	339.5
	*glucan1,3-beta-glucosidase I/II*	299.3				
	ribonuclease T2-like	299				
TD70	*invertase 2*	551.9	*glucan1,3-beta-glucosidase*	663.1	phosphatidylglyce-rol/phosphatidylinositol transfer protein	279.5
	*1,3-beta-glucanosyltransfer-aseGas1p*	410.5	cerevisin	501.1		
	*lysophospholipase 1*	394.7	*invertase 2*	392.5		
	*probable1,3-beta-glucanosyltransfer-aseGas3p*	489.9	NPC intracellular sterol transporter 1-related protein 1	444.6		
	aminopeptidase Y	422.5	*protein Ygp1p*	431.9		
	carboxypeptidase Y	434	*cell wall mannoprotein Cis3p*	340.6		
	*cell wall protein Ecm33p*	262.2				
	*probable glycosidase Crh1p*	377.8				

a Proteins identified within each MW range for the different MPs are ranked according to |Log Prob| to which a cut-off value of 4 was applied. Glycosylated CW proteins have been italicized.

b MW range of the excised protein bands as indicated in Figure 5.

c Name of the identified protein according to UniProt.

d The largest Byonic score of a PSM for the given protein, which is the primary indicator of PSM correctness.

Although CW MPs were identified in almost all excised bands, these were not the only identified proteins nor the proteins with the highest |Log Prob| or largest Byonic score for a PSM in any of the samples analyzed. Nevertheless, acceptable Byonic scores of >300 were obtained for the highest-scoring PSMs of the most identified MPs. In SB62 and SC01, six unique CW MPs were identified across all three MW ranges, whereas two were identified in TD70 and one in MF77. Of the three MPs identified in the 40–70 kDa region for SB62, two were unique to this MW range for SB62 (Ccw14p and “GPI-anchored protein”), whereas two identified MPs were unique to the 20–30 kDa range (Cwp1p and Tos1p) and one to the 10–20 kDa (Hsp150p) excised region, in which three and two MPs had been identified, respectively. In the 40–70, 20–30, and 10–20 kDa regions of SC01, four, two, and four MPs were identified, respectively, of which Ccw14p and EC1118_1J11_0650p were unique to the 40–70 kDa excised band and Hsp150p and Hpf1p were unique in the 10–20 kDa range. Cis3p was identified in all MF77 bands analyzed and also in the 20–30 kDa region of TD70. A MP in the 40–70 kDa region, Ecm33p, was uniquely identified in TD70 (Figure 6). Other MPs unique to the yeast species of this study include Tos1p and GPI-anchored proteins in SB62 and EC1118_1J11_0650p and Hpf1p in SC01. SB62 and SC01 furthermore shared three MPs that had not been identified in the other two species, namely, Ccw14p, Cwp1p, and Hsp150p. All four species shared the CW MP Cis3p. Other CW glycoproteins that were shared by all four yeasts included invertases (saccharase), 1,3-beta-glucanosyltransferases, 1,3-beta-glucosidases, asparaginases, glycosidases, and lysophospholipases.

Other proteins were also identified in the bands excised for analysis from all of the species (Table 2). These included intracellular proteins such as GDH, actin, triosephosphate isomerase, carboxypeptidase Y, ribonuclease T2, cerevisin, and PITP and extracellular enzymes including invertase and several peptide hydrolases.

## Discussion

This research investigated some of the compositional differences that exist between MPs isolated from different yeast species, particularly in terms of their protein profiles, mannose/glucose ratios, polysaccharide size distribution, and protein/carbohydrate ratios. MP-rich preparations from SB62, SC01, MF77, and TD70 were obtained through ultrasound and β-1,3-glucanase treatment based on the method optimized previously.^21^ A commercial MP formulation, LMP, was used for comparison purposes. Important differences were observed between these MPs and LMP, particularly in terms of the mannose/glucose ratios and the polysaccharide size distribution profile. Compared to the other MPs, the percentage of mannose was 9–18% reduced in LMP, and the weighted average MW of polysaccharides was lower. Although these variations could be strain-dependent, it is also possible that they are due to the different extraction methods followed for the commercial MP and the other MPs of this study. Indeed, the MP structure and composition have been shown to be influenced not only by the yeast species of origin but also by the methods used to extract them from the CW and isolate or purify them.^3,26^ Furthermore, it is possible that some oligosaccharide families that were excluded from the ConA-purified samples were still present in LMP, which could explain the differences in size distribution patterns detected by HRSEC analysis (Figure 4).

It is clear from the PID analysis that CW MPs were not the only proteins present in ConA-purified fractions (Table 2). The presence of intra- and extracellular proteins indicates that the combined ultrasound and β-glucanase extraction method used for SB62, SC01, MF77, and TD70 was not specific for the extraction of CW compounds. The shear forces generated by the sonication parameters employed in this study likely ruptured not only the CWs of the yeast but also led to the subsequent disruption of the resultant protoplasts, thus partially releasing their intracellular contents.^27^ It is furthermore likely that the centrifugation steps of the extraction method led to the sedimentation and inclusion of not only the cellular components but also extracellular proteins that were secreted during culturing. The identification of non-MP proteins also called into question the specificity of ConA for MPs. In fact, although ConA shows greater preference for highly mannosylated molecules with a large degree of branching such as MPs, its affinity for α-linked mannose residues allows it to bind many yeast glycoproteins as all N-linked glycans are modified with mannose.^28−30^ Furthermore, 20–50% of yeast proteins are estimated to be glycosylated to various degrees; thus, it is to be expected that the extraction of both extra- and intracellular proteins in addition to CW proteins would also lead to the purification of glycoproteins other than CW MPs.^28^ Indeed, many of the proteins with high |Log Prob| values identified in this study, such as invertase, 1,3-beta-glucanosyltransferase, 1,3-beta-glucosidase, asparaginase, lysophospholipase, ribonuclease, several peptide hydrolases and glycosidases, cerevisin, and permease, are post-translationally modified with a glycan group (information obtained from UniProt). It would therefore seem that although MPs were not the only compounds separated by ConA, this method did allow the enrichment of yeast glycoproteins. This can furthermore be observed in the higher sugar yields obtained from the crude extract after ConA purification compared to the protein yields and the higher ratio of sugar to protein in purified samples compared to the crude extracts (Figure 2b,c).

Despite the presence of other proteins in the ConA-purified samples, MPs were identified in almost all of the deglycosylated protein bands submitted for LC–MS/MS sequencing. Many of the identified proteins, MPs and otherwise, were shared between species (Figure 6). Indeed, this is reflected in the similarities observed between protein profiles obtained by PAGE under both native and denaturing conditions with and without glycosylation, respectively (Figures 1a and 5). The low relative mobility of the protein bands observed after native PAGE is likely due to the bulky nature of the glycosylated proteins which allowed limited migration through the gel, the carbohydrate moiety of which could be visualized after PAS staining at >250 kDa. The protein banding patterns of SB62 and SC01 in particular show great similarity, which can furthermore be correlated with the larger number of proteins shared exclusively between these two species, compared with any of the other two species. Nevertheless, some of the protein bands in common between species, specifically those with MWs of ∼26, ∼14, and ∼12 kDa, could be due to the leakage of ConA monomers and fragments, particularly when proteins were electrophoresed under denaturing conditions, as previously reported.^31^ This leakage of ConA, resulting in the migration of fragments across different MW ranges, could furthermore be partially responsible for the identification of similar proteins in multiple MW ranges, should they remain associated with the lectin (Table 2).

Most of the shared MP identifications were between SC01 and SB62, which had Ccw14p, Cwp1p, and Hsp150p in common to the exclusion of MF77 and TD70 (Figure 6). The CW MP Cis3p was shared between all species, whereas some MPs were identified exclusively to certain species. Specifically, Tos1p and “GPI-anchored protein” were only found in SB62, whereas EC1118_1J11_0650p and Hpf1p were identified in SC01 and Ecm33p in TD70. However, many of these MPs belong to similar protein families. Cis3p and Hsp150p both belong to the PIR (proteins with internal repeats) protein family, which is characterized by the covalent linkage between glutamine residues of their repetitive sequences directly to glucose of CW β-1,3-glucan.^32^ The expression of PIR genes is upregulated under conditions of CW stress, suggesting their role in CW strengthening.^33^ On the other hand, Ecm33p and “GPI-anchored protein” both belong to the Sps2 protein family, which constitutes a group of glycosyl phosphatidyl inositol (GPI)-dependent CW proteins linked to β-1,6-glucan via a GPI remnant and may be localized to the CW or the plasma membrane.^34^ Ecm33p in particular is important for CW integrity, and various GPI–CWPs (cell wall proteins) are involved in adhesion events such as sexual agglutination and flocculation.^35^ Other protein families describing GPI–CWPs include, among others, the Pga52 family, which constitutes both Tos1p and EC1118_1J11_0650p identified in this study; the Srp1/Tip1 family, which includes Cwp1p and Hpf1p; and the Ccw14 protein family.^34,36,37^ Several of the MPs identified in this research have, in previous studies, been associated with important roles in wine quality. For example, Ccw14p (identified in SB62 and SC01) has been shown to promote the process of flor formation.^38^ Flor production has also been associated with the upregulation of the genes encoding the two PIR proteins identified in this study, namely, Hsp150p and Cis3p, of which the latter is the MP common to all four species of this study.^39^ Furthermore, the protein Hpf1p, identified in SC01 in this study, has been shown to reduce protein haze in white wine, possibly through interactions with haze-forming proteins and/or other wine macromolecules involved in haze formation, such as wine polyphenols.^9,40^ It remains to be investigated whether Cwp1p, which shares a protein family with Hpf1p, and Ecm33p, identified in TD70 and which shares a sequence similarity of 58% with Hpf1p, also contribute similar haze-protective benefits to white wine.^41^ Several glycosylated cell wall proteins with known and predicted enzymatic activities, such as invertases (saccharase), 1,3-beta-glucanosyltransferases, 1,3-beta-glucosidases, asparaginases, glycosidases, and lysophospholipases, were furthermore shared between all four species. The presence of enzymes such as β-glucanase has also been reported for some commercial MP preparations, likely due to limited degrees of purification.^48^ However, when considering the PID analyses of this study and attempting to compare the different species used, it is imperative to note the discrepancies in annotation completeness that exist between the proteome databases available for the strains in question. Due to these discrepancies, the interrogation of a *S. cerevisiae* reference proteome was made for the identification of proteins from *M. fructicola* and *T. delbrueckii,* which likely led to the loss of much information due to nonhomologous sequences between species. It would therefore be of great interest to repeat this interrogation against the annotated proteome databases of *M. fructicola* and *T. delbrueckii* when they become available in the future.

Further investigations were made into the polysaccharide composition of the ConA-purified preparations in an attempt to characterize the glycan moiety of the MPs from SB62, SC01, MF77, and TD70. Monosaccharide analysis, specifically of glucose and mannose composition, revealed increasing mannose/glucose ratios of 15.9–52.1 for the different species in the order of MF77 < TD70 < SC01 < SB62 where the ratio for SB62 was more than twice that of SC01, which in turn showed a 1.5-fold increase compared to MF77 and TD70. This may be relevant to the potential influence of these compounds on wine properties, such as haze reduction and the modulation of tannin aggregation.^13,14,42^ Previous studies have demonstrated the positive effect of MPs with higher mannose-to-glucose ratios on wine protein stabilization and the prevention of haze formation, whereas a lower ratio has been associated with the modulation of tannin aggregation.^13,14,42^ Possibly these compositional differences modify the spatial conformation of the MP, thus influencing its colloidal behavior.^43^ However, the reported mannose/glucose ratios in MPs are typically much lower than those found in this study, ranging from 0.61 to 10.^14,43^ It is possible that the presence of heavily mannosylated glycoproteins, in addition to CW MPs, in the ConA-purified preparation contributed to this ratio. Nevertheless, the possibility of contaminating glucan residues present in the MP preparations contributing to the glucose concentrations should be considered.

Size exclusion chromatography of polysaccharides contained in the purified fractions furthermore revealed similar size distribution patterns but with some differences regarding the MW range covered by polysaccharides and the relative proportion of polysaccharides compared to oligosaccharides. The higher levels of oligosaccharides in SC01 and MF77 are possibly due to heightened levels of glycoside hydrolases such as mannosidases, resulting in the digestion of the polysaccharide moiety of MP and the production of lower MW oligosaccharides. Indeed, as discussed previously, it was evident from PID analyses that many hydrolytic enzymes had been extracted and purified alongside MPs. It is also possible that the extraction method itself led to the partial hydrolyzation of all MPs.^44^ Furthermore, while all polysaccharides showed a similar minimum MW of ∼5 kDa, those of MF77 reached up to 1349 kDa while SB62 and SC01 showed maximum MWs of 1014 and 1022 kDa, and TD70 displayed the smallest polysaccharides with an upper limit of 483 kDa. Nevertheless, the majority of the polysaccharides for all species in this study fall in the range of 67.7–78.4 kDa, which, although within the typical range of 50–500 kDa found for most MPs in wine, is considered relatively small.^45,46^ Previous studies have demonstrated the influence of MP MW on the stabilization effect of polyphenols in wine.^12,16,47^ Specifically, lower MW MPs were more efficient at protecting polyphenol aggregates from precipitation, possibly through a steric stabilizing mechanism.

The ratio of sugar to protein contained within a MP can give further valuable information regarding its composition and function. In this study, sugar/protein ratios of 14.7, 12.1, 13.9, and 15.6 were recorded for SB62, SC01, MF77, and TD70, respectively. The protein percentages, at 5.0–6.5%, were slightly higher than the range of 2.4–3.9% observed for non-*Saccharomyces* MPs in previous studies.^19,20^ This could be due to the additional glycoproteins purified alongside MPs, as discussed earlier. The significance of the proportion of carbohydrate to protein in MPs to wine quality has been noted in terms of both haze protection and the interaction of salivary proteins with wine flavanols.^5,6^ Indeed, it has been suggested that glycosylation could provide an active site for the interaction of MPs with wine components such as haze-forming proteins and that an elevated glycan content allowed the stabilization of soluble aggregates with salivary proteins and phenolic compounds, which have implications for wine astringency.

In conclusion, several differences in the composition of the MP-rich preparations between the different wine yeast strains of this study could be observed, such as the mannose-to-glucose ratio and the range of the polysaccharide size distribution. Previous studies have demonstrated the importance of these properties to elements of wine quality such as protein stability and astringency; however, the impact of the MPs from this study on wine remains to be established. Some of the similarities observed between MPs could be related to the common protein families to which they belong, possibly indicating similar glycosylation patterns and linkages and associations with the CW. Nevertheless, a serious limitation to a thorough PID analysis was presented by the lack of well-annotated proteome databases for MF77 and TD70; thus, a repetition of this analysis in the future may yield more valuable information. Further work should also evaluate the content of additional monosaccharides such as galactose and galacturonic acid, the levels of which have been shown to vary between yeast species. Lastly, some of the largest differences observed in this study were between the commercial LMP and those extracted and purified in this study. Extraction and purification methods have been shown to influence the structure and composition of MPs, which, in turn, have an impact on their function. When MPs are compared, it is therefore important to consider not only pertinent differences such as their yeast strain of origin but also the method by which they were obtained in order to establish a more comprehensive view of the elements that contribute to their structure and, ultimately, their impact on wine properties. Future work may therefore include the use of a commercially available enological yeast strain as a control for extraction and purification alongside those used in this study, instead of a commercial MP prepared through different methods.

## Supporting Information Available

Tabular summary of additional information regarding protein identification analyses, including the UniProt entry names, |Log Prob| values, number of unique peptides, and percentage coverage (PDF)

## Abbreviations Used

MP: mannoprotein
PIR: protein with internal repeats
GPI: glycosylphosphatidylinositol
FPLC: fast protein liquid chromatography
ConA: concanavalin A
SDS–PAGE: sodium dodecyl sulfate polyacrylamide gel electrophoresis
APS: ammonium persulfate
TEMED: *N*,*N*,*N*′,*N*′-tetramethylethylenediamine
PAS: periodic acid-Schiff
GC–FID: gas chromatography–flame ionization detection
TFA: trifluoroacetic acid
TMS: trimethylsilyl
MW: molecular weight
HRSEC: high-resolution size-exclusion chromatography
RID: refractive index detection
PID: protein identification
LC–MS/MS: liquid chromatography with tandem mass spectrometry
PSM: peptide spectrum match
MWCO–: molecular weight cutoff
kDa: kilodalton
GDH: glutamate dehydrogenase
CW: cell wall
TPI: triosephosphate isomerase
GAPDH: glyceraldehyde-3-phosphate dehydrogenase
PITP: phosphatidylglycerol/phosphatidylinositol transfer protein
