# Supplementary material for: Characterization of Mannoprotein Structural Diversity in Wine Yeast Species
Source: J Agric Food Chem. 2023 Dec 4;71(49):19727–38. doi: 10.1021/acs.jafc.3c05742 (PMC10722544; doi:10.1021/acs.jafc.3c05742)
Supplement: Supplementary file 1 — jf3c05742_si_001.pdf [file jf3c05742_si_001.pdf]

Supporting Information

Characterisation of Mannoprotein Structural Diversity in Wine Yeast Species

Carla Snyman[1,2], Julie Mekoue Nguela[3], Nathalie Sieczkowski[3], Benoit Divol[1,*] and Matteo Marangon[2,4]

[1]South African Grape and Wine Research Institute, Department of Viticulture and Oenology, Stellenbosch University, Private Bag X1, Matieland 7602, South Africa

[2]Department of Agronomy, Food, Natural Resources, Animals and Environment (DAFNAE), University of Padova, Viale Dell'Università, 16, 35020, Legnaro, Padova, Italy

[3]Lallemand SAS, 19 rue des briquetiers, BP 59, 31702 Blagnac, France

[4] Interdepartmental Centre for Research in Viticulture and Enology (CIRVE), University of Padova, via XXVIII Aprile 14, 31015 Conegliano, Italy

*Corresponding Author:
E-mail: divol@sun.ac.za
Tel: (+27) 21 808 3141

Table S1. Proteins identified through peptide spectrum matches (PSMs) using tandem mass spectrometry and Byonic software in MPs purified from *S. boulardii* (SB62), *S. cerevisiae* (SC01), *M. fucticola* (MF77) and *T. delbrueckii* (TD70). Proteins identified within each MW range for the different MPs are ranked according to |Log Prob| to which a cut-off value of 4 was applied. Glycosylated cell wall proteins have been highlighted in light grey. [a] Molecular weight (MW) range of the excised protein bands as indicated in Figure 5. [b] Name of the identified protein according to UniProt. [c] Mnemonic identifier for the protein entry on UniProtKB. [d] Log base 10 of the protein p-value which is the likelihood of the PSMs to this protein (or protein group) arising by random chance. [e] The largest Byonic score of a PSM for the given protein, which is the primary indicator of PSM correctness. [f] The total number of PSMs for the given protein, excluding duplicates. [g] Percent of the protein sequence covered by PSMs.

Sample	MW range[a] (kDa)	Protein name[b]	UniProt entry name[c]	|Log Prob|[d]	Best score[e]	# of unique pep-tides[f]	Cover-age %[g]
**SB62**	40 - 70	Peptide hydrolase	A0A0L8VV61_9SACH	151.52	783.70	29	62.38
		Saccharase	A0A0L8VNE4_9SACH	117.47	649.00	27	41.99
		1,3-beta-glucanosyltransferase	A0A0L8VJC3_9SACH	105.52	751.50	19	45.99
		1,3-beta-glucanosyltransferase	A0A0L8VJZ4_9SACH	84.19	531.60	20	34.53
		Glycosidase	A0A0L8VQQ6_9SACH	83.43	785.20	17	24.70
		APE1p AminoPeptidase	A0A0L8VMY5_9SACH	77.01	853.70	18	33.46
		Lysophospholipase	A0A0L8VJY8_9SACH	36.73	562.20	11	15.05
		Bgl2p Endo-beta-1,3-glucanase	A0A0L8VPN7_9SACH	35.64	610.40	7	22.36
		Alpha-mannosidase	A0A0L8VQQ1_9SACH	31.40	429.10	10	9.42
		Ccw14p Covalently linked cell wall glycoprotein	A0A0L8VLP4_9SACH	23.25	644.10	6	18.07
		Ygp1p Cell wall-related secretory glycoprotein	A0A0L8VJD0_9SACH	19.42	460.40	4	11.58
		Glutamate dehydrogenase (GDH)	A0A0L8VHH8_9SACH	18.81	390.80	6	14.76

	Cis3p Mannose-containing glycoprotein constituent of the cell wall	A0A0L8VMU4_9SACH	12.78	545.00	4	11.56
	GPI-anchored protein	A0A0L8VV35_9SACH	9.32	588.20	3	7.69
	1,3-beta-glucanosyltransferase	A0A0L8VHM7_9SACH	5.81	438.90	2	4.75
20 -30	Bgl2p Endo-beta-1,3-glucanase	A0A0L8VPN7_9SACH	126.54	946.50	24	69.65
	Peptide hydrolase	A0A0L8VV61_9SACH	82.32	792.60	18	47.86
	Saccharase	A0A0L8VNE4_9SACH	62.16	563.70	16	29.49
	Glycosidase	A0A0L8VQQ6_9SACH	56.75	684.40	15	23.51
	Ygp1p Cell wall-related secretory glycoprotein	A0A0L8VJD0_9SACH	35.62	553.40	7	21.75
	Lysophospholipase	A0A0L8VJY8_9SACH	33.76	537.50	7	13.37
	Cwp1p Cell wall mannoprotein that localizes to birth scars of daughter cells	A0A0L8VM50_9SACH	24.24	512.20	7	35.15
	Prb1p Vacuolar proteinase B (YscB) with H3 N-terminal endopeptidase activity	A0A0L8VS42_9SACH	21.31	501.70	5	7.09
	Cis3p Mannose-containing glycoprotein constituent of the cell wall	A0A0L8VMU4_9SACH	20.01	633.10	7	13.78
	Tos1p Covalently-bound cell wall protein	A0A0L8VV43_9SACH	19.38	626.90	3	14.51
	1,3-beta-glucanosyltransferase	A0A0L8VJZ4_9SACH	16.29	383.00	6	12.88
	1,3-beta-glucanosyltransferase	A0A0L8VJC3_9SACH	16.02	515.20	5	14.31

		Triosephosphate isomerase (TPI)	A0A0L8VSZ3_9SACH	10.76	301.50	3	16.13
		1,3-beta-glucanosyltransferase	A0A0L8VHM7_9SACH	6.49	351.30	2	5.58
		Ape4p Cytoplasmic aspartyl aminopeptidase with possible vacuole function	A0A0L8VPD5_9SACH	5.82	315.80	3	8.92
		Phosphatidylglycerol/phosphatidylinositol transfer protein	A0A0L8VSK6_9SACH	5.06	229.10	2	13.29
	10 - 20	Bgl2p Endo-beta-1,3-glucanase	A0A0L8VPN7_9SACH	25.91	547.90	11	35.14
		Saccharase	A0A0L8VNE4_9SACH	19.62	375.70	12	23.24
		Cis3p Mannose-containing glycoprotein constituent of the cell wall	A0A0L8VMU4_9SACH	9.99	465.90	4	11.56
		Lysophospholipase	A0A0L8VJY8_9SACH	8.84	304.40	6	9.12
		Peptide hydrolase	A0A0L8VV61_9SACH	8.30	485.10	4	10.06
		Hsp150p O-mannosylated heat shock protein	A0A0L8VNF4_9SACH	5.74	545.00	2	4.36
		Phosphatidylglycerol/phosphatidylinositol transfer protein	A0A0L8VSK6_9SACH	4.96	282.00	3	16.76
		1,3-beta-glucanosyltransferase	A0A0L8VJC3_9SACH	4.77	420.70	3	5.92
		Actin	A0A0L8VR24_9SACH	4.67	280.50	4	11.47
		Glycosidase	A0A0L8VQQ6_9SACH	4.24	265.70	5	6.97
**SC01**	40 -70	Saccharase	C8ZAV0_YEAS8	76.03	598.90	21	37.15
		Peptide hydrolase	D3UF33_YEAS8	72.61	674.00	14	37.06
		Pep4p (Proteinase)	C8ZIM4_YEAS8	65.84	891.20	14	30.37

	1,3-beta-glucanosyltransferase	C8ZFH7_YEAS8	65.20	496.20	23	36.85
	Cwp1p (Cell wall protein)	C8ZC79_YEAS8	53.96	734.50	11	45.19
	Bgl2p (Glucan 1,3-beta-glucosidase)	C8Z9H5_YEAS8	46.74	710.10	13	32.59
	Ygp1p (Asparaginase)	C8ZG69_YEAS8	45.30	557.90	12	32.77
	1,3-beta-glucanosyltransferase	C8ZF79_YEAS8	45.21	678.30	11	30.34
	Carboxypeptidase	C8ZFG6_YEAS8	30.06	599.00	7	17.11
	Lysophospholipase	C8ZEL4_YEAS8	28.89	540.90	7	12.01
	EC1118_1J11_0650p (Cell wall protein)	C8ZB48_YEAS8	26.87	455.30	5	18.69
	Prb1p (Proteinase)	C8Z6T1_YEAS8	20.48	532.60	7	10.55
	Glycosidase	C8Z982_YEAS8	19.48	472.30	7	12.95
	Exg1p (Glucan 1,3-beta-glucosidase)	C8ZDR3_YEAS8	18.52	456.60	7	20.76
	1,3-beta-glucanosyltransferase	C8ZHT4_YEAS8	17.10	509.40	5	12.79
	Ccw14p (Covalently linked cell wall protein)	C8ZE00_YEAS8	12.99	670.70	3	19.03
	Cis3p (Cell wall mannoprotein)	C8ZB06_YEAS8	9.91	553.30	4	11.56
	Lap4p (Aminopeptidase)	C8ZC72_YEAS8	9.74	319.00	3	7.20
	Ecm14p (Peptidase)	C8Z9S6_YEAS8	6.45	295.70	3	9.77
	Alpha-mannosidase	C8Z895_YEAS8	4.40	296.90	2	2.22
20 -30	Pep4p (Proteinase)	C8ZIM4_YEAS8	135.73	774.80	19	59.75
	Bgl2p (Glucan 1,3-beta-glucosidase)	C8Z9H5_YEAS8	126.63	1033.80	21	66.13
	Cwp1p (Cell wall protein)	C8ZC79_YEAS8	119.98	830.70	16	45.19
	Peptide hydrolase	D3UF33_YEAS8	107.51	771.50	14	37.06
	Saccharase	C8ZAV0_YEAS8	98.21	632.30	20	28.71

	1,3-beta-glucanosyltransferase	C8ZF79_YEAS8	77.28	664.10	13	33.78
	Ygp1p (Asparaginase)	C8ZG69_YEAS8	71.40	698.10	12	24.01
	Prb1p (Proteinase)	C8Z6T1_YEAS8	59.44	817.30	12	22.83
	Exg1p (Glucan 1,3-beta-glucosidase)	C8ZDR3_YEAS8	47.27	579.40	9	22.32
	1,3-beta-glucanosyltransferase	C8ZFH7_YEAS8	34.63	539.80	8	16.99
	Lysophospholipase	C8ZEL4_YEAS8	25.52	641.50	7	15.81
	Cis3p (Cell wall mannoprotein)	C8ZB06_YEAS8	25.30	728.70	4	11.56
	1,3-beta-glucanosyltransferase	C8ZHT4_YEAS8	23.68	624.30	4	10.27
	Glycosidase	C8Z982_YEAS8	23.08	554.20	7	8.96
	Tfs1p (Carboxypeptidase inhibitor)	C8ZDF0_YEAS8	19.23	416.90	5	34.70
	Glyceraldehyde-3-phosphate dehydrogenase	C8Z985_YEAS8	19.00	639.00	7	22.59
	Carboxypeptidase	C8ZFG6_YEAS8	17.93	471.00	5	15.79
	Adp1p (Permease)	C8Z498_YEAS8	11.88	344.20	3	3.24
	Ribonuclease T(2)	C8ZIQ6_YEAS8	11.56	482.00	5	15.44
	Phosphatidylglycerol/phosphatidylinositol transfer protein	C8Z4M3_YEAS8	7.62	512.50	2	9.83
10 -20	Pep4p (Proteinase)	C8ZIM4_YEAS8	104.75	852.90	14	51.60
	Bgl2p (Glucan 1,3-beta-glucosidase)	C8Z9H5_YEAS8	102.59	899.70	14	58.15
	Cwp1p (Cell wall protein)	C8ZC79_YEAS8	89.26	810.60	13	45.19
	Saccharase	C8ZAV0_YEAS8	89.25	596.10	15	28.33
	Peptide hydrolase	D3UF33_YEAS8	62.43	644.60	9	23.09

		1,3-beta-glucanosyltransferase	C8ZF79_YEAS8	58.36	731.90	8	22.90
		Ygp1p (Asparaginase)	C8ZG69_YEAS8	49.99	686.20	10	21.47
		1,3-beta-glucanosyltransferase	C8ZFH7_YEAS8	49.94	561.80	11	24.33
		Lysophospholipase	C8ZEL4_YEAS8	41.28	671.90	7	12.92
		Prb1p (Proteinase)	C8Z6T1_YEAS8	40.97	694.50	8	12.28
		Exg1p (Glucan 1,3-beta-glucosidase)	C8ZDR3_YEAS8	31.28	609.90	6	20.54
		Cruciform DNA-recognizing protein 1	CRP1_YEAS8	31.01	586.30	5	9.46
		Glycosidase	C8Z982_YEAS8	26.35	483.00	9	11.35
		Cis3p (Cell wall mannoprotein)	C8ZB06_YEAS8	24.87	841.90	5	11.56
		EC1118_1H13_1101p (Uncharacterised protein)	C8Z9T4_YEAS8	20.53	523.90	3	37.72
		1,3-beta-glucanosyltransferase	C8ZHT4_YEAS8	20.25	580.30	3	8.39
		Phosphatidylglycerol/phosphatidylinositol transfer protein	C8Z4M3_YEAS8	18.97	494.10	5	32.37
		Hsp150p (Cell wall mannoprotein)	C8ZB60_YEAS8	11.92	460.00	3	4.57
		Carboxypeptidase	C8ZFG6_YEAS8	6.87	454.60	2	3.57
		Hpf1p (Haze protective factor)	C8ZHG3_YEAS8	5.90	319.30	2	6.83
**MF77**	40 - 70	Invertase 2	INV2_YEAST	65.53	491.00	26	32.52
		1,3-beta-glucanosyltransferase GAS1	GAS1_YEAST	35.41	481.90	20	31.66
		Probable 1,3-beta-glucanosyltransferase Gas3p	GAS3_YEAST	32.72	529.10	8	18.70
		Probable glycosidase Crh1p	CRH1_YEAST	29.05	414.70	10	16.96

		Glucan 1,3-beta-glucosidase	BGL2_YEAST	23.75	486.20	9	20.13
		Lysophospholipase 1	PLB1_YEAST	17.66	428.10	10	14.16
		Aminopeptidase Y	APE3_YEAST	11.68	441.00	6	11.92
		Cell wall mannoprotein Cis3p	CIS3_YEAST	9.24	423.90	4	11.45
		Glucan 1,3-beta-glucosidase I/II	EXG1_YEAST	5.38	299.30	2	5.58
		Ribonuclease T2-like	RNY1_YEAST	4.45	299.00	4	12.90
	20 - 30	Glucan 1,3-beta-glucosidase	BGL2_YEAST	67.85	700.50	13	38.98
		Invertase 2	INV2_YEAST	47.00	466.00	14	25.19
		Cerevisin	PRTB_YEAST	22.28	615.30	7	9.92
		Probable glycosidase CRH1	CRH1_YEAST	12.87	395.00	6	7.69
		Cell wall mannoprotein Cis3p	CIS3_YEAST	8.45	445.70	3	11.45
		Protein Ygp1p (Asparaginase)	YGP1_YEAST	8.15	402.30	2	5.93
		Aminopeptidase Y	APE3_YEAST	5.67	368.70	3	5.96
		Lysophospholipase 1	PLB1_YEAST	5.59	395.80	2	4.97
	10 -20	Glucan 1,3-beta-glucosidase	BGL2_YEAST	21.17	461.20	5	22.36
		Invertase 2	INV2_YEAST	20.91	386.90	7	12.03
		Aminopeptidase Y	APE3_YEAST	17.17	536.00	6	13.41
		Lysophospholipase 1	PLB1_YEAST	17.01	688.80	4	7.53
		Probable 1,3-beta-glucanosyltransferase Gas3p	GAS3_YEAST	11.03	470.90	3	5.92
		Phosphatidylglycerol/phosphatidylinositol transfer protein	NPC2_YEAST	10.05	403.70	3	16.76
		1,3-beta-glucanosyltransferase Gas1p	GAS1_YEAST	7.94	364.10	3	6.26
		Cell wall mannoprotein Cis3p	CIS3_YEAST	7.04	339.50	3	11.45
**TD70**	40 -70	Invertase 2	INV2_YEAST	59.80	551.90	19	34.21

	1,3-beta-glucanosyltransferase Gas1p	GAS1_YEAST	31.62	410.50	11	23.97
	Lysophospholipase 1	PLB1_YEAST	24.55	394.70	7	12.80
	Probable 1,3-beta-glucanosyltransferase Gas3p	GAS3_YEAST	21.03	489.90	6	13.36
	Aminopeptidase Y	APE3_YEAST	16.60	422.50	9	22.53
	Carboxypeptidase Y	CBPY_YEAST	16.36	434.00	7	11.09
	Cell wall protein Ecm33p	ECM33_YEAST	5.56	262.20	2	5.13
	Probable glycosidase Crh1p	CRH1_YEAST	5.19	377.80	4	5.92
20 - 30	Glucan 1,3-beta-glucosidase	BGL2_YEAST	54.86	663.10	11	38.98
	Cerevisin	PRTB_YEAST	19.64	501.10	5	7.72
	Invertase 2	INV2_YEAST	19.40	392.50	7	12.41
	NPC intracellular sterol transporter 1-related protein 1	NPC1_YEAST	6.25	444.60	2	2.05
	Protein Ygp1p	YGP1_YEAST	5.60	431.90	2	5.93
	Cell wall mannoprotein Cis3p	CIS3_YEAST	4.95	340.60	2	7.93
10 - 20	Phosphatidylglycerol/phosphatidylinositol transfer protein	NPC2_YEAST	4.62	279.50	2	13.29
